# FunOrder 2.0 – a method for the fully automated curation of co-evolved genes in fungal biosynthetic gene clusters

**DOI:** 10.3389/ffunb.2022.1020623

**Published:** 2022-10-25

**Authors:** Gabriel A. Vignolle, Robert L. Mach, Astrid R. Mach-Aigner, Christian Zimmermann

**Affiliations:** ^1^ Institute of Chemical, Environmental and Bioscience Engineering, TU Wien, Vienna, Austria; ^2^ Center for Health & Bioresources, Competence Unit Molecular Diagnostics, AIT Austrian Institute of Technology GmbH, Vienna, Austria

**Keywords:** coevolution, secondary metabolism, fungi, ascomycetes, bioinformatics, genome mining

## Abstract

Coevolution is an important biological process that shapes interacting proteins – may it be physically interacting proteins or consecutive enzymes in a metabolic pathway, such as the biosynthetic pathways for secondary metabolites. Previously, we developed FunOrder, a semi-automated method for the detection of co-evolved genes, and demonstrated that FunOrder can be used to identify essential genes in biosynthetic gene clusters from different ascomycetes. A major drawback of this original method was the need for a manual assessment, which may create a user bias and prevents a high-throughput application. Here we present a fully automated version of this method termed FunOrder 2.0. In the improved version, we use several mathematical indices to determine the optimal number of clusters in the FunOrder output, and a subsequent k-means clustering based on the first three principal components of a principal component analysis of the FunOrder output to automatically detect co-evolved genes. Further, we replaced the BLAST tool with the DIAMOND tool as a prerequisite for using larger proteome databases. Potentially, FunOrder 2.0 may be used for the assessment of complete genomes, which has not been attempted yet. However, the introduced changes slightly decreased the sensitivity of this method, which is outweighed by enhanced overall speed and specificity.

## Introduction

Every form of life known to humankind is subjected to evolution. This process shapes and forms all biological systems on the macroscopic and molecular levels. Thus, understanding and detecting evolutionary processes substantially contributes to understanding life forms and life itself. An important evolutionary process is the so-called coevolution. This is defined as a “process of reciprocal evolutionary change that occurs between pairs of species or among groups of species as they interact with one another” (Rafferty and Thompson). This definition can be extended to interacting proteins (Fraser et al., 2004), may it be physical interactions, or may it be consecutive actions in a metabolic pathway. Thus, interacting proteins and the corresponding genes can be assumed to have a similar evolutionary history.

**Figure 1:**
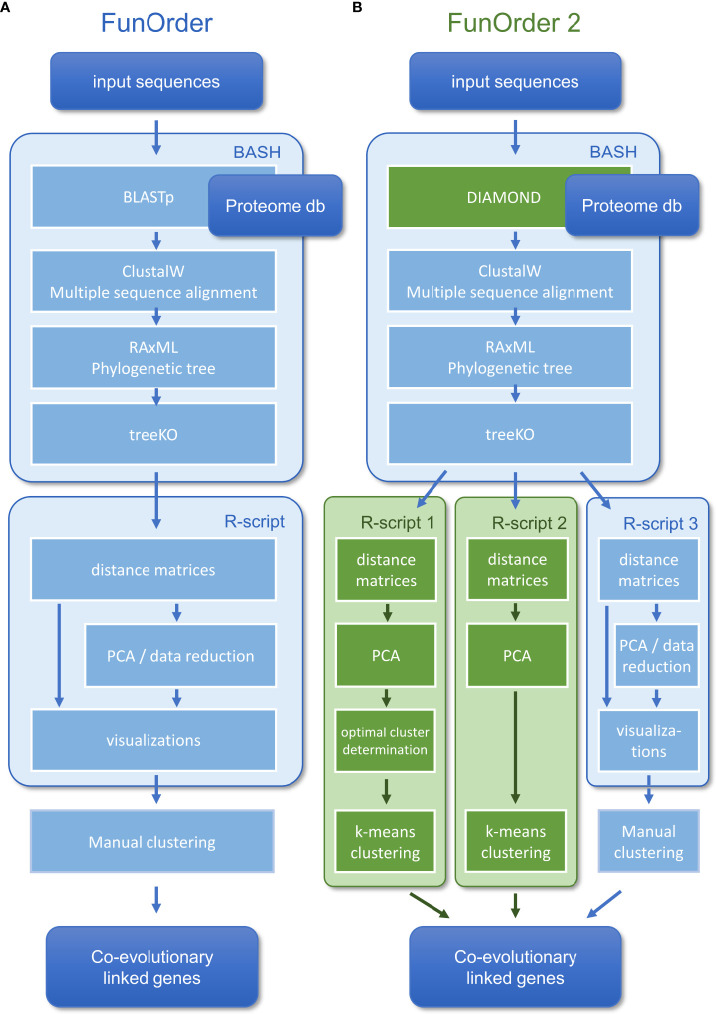
Comparison of the workflow of the original FunOrder method **(A)** and FunOrder 2.0 **(B)**.

In a previous study, we described a semi-automated method for the identification of coevolutionary linked genes, named FunOrder (Vignolle et al., 2021). Therein, the protein sequences of an input set of proteins are blasted against an empirically optimized proteome database. The Top 20 results of each search are then compared in a multisequence alignment, and a phylogenetic tree is calculated for each input protein. Next, the phylogenetic trees of all proteins are compared pairwise using the treeKO tool. This tool calculates how similar two trees are, and thus how similar the evolutionary history of two proteins is. The treeKO tool calculates two distances, the strict distance, and the speciation distance. Notably, the strict distance had previously been suggested to be more suitable for the detection of coevolution in protein families than the speciation (or evolutionary) distance (Marcet-Houben and Gabaldon, 2011). Further, we combined the two distance values to a third measure, the combined distance, to consider also the speciation history in the FunOrder method. The strict and the combined distances of all pairwise comparisons were then compiled in two matrices and visualized as heatmaps, dendrograms, and two principal component analyses (PCA) were performed. In the final step of this method, the user needed to assess these different visualizations of the underlying data to detect co-evolved proteins (**Figure 1A**). Please refer to the original study for a detailed description of this method (Vignolle et al., 2021).

In the previous publication, we demonstrated the functionality and applicability of this method by identifying essential genes in biosynthetic gene clusters (BGCs) of ascomycetes (Vignolle et al., 2021). Fungal BGCs contain genes whose corresponding enzymes constitute metabolic pathways for the biosynthesis of secondary metabolites (SMs) (Osbourn, 2010). SMs are a vast group of compounds with different structures and properties that are not necessary for the normal growth of an organism but can be beneficial under certain conditions (Keller et al., 2005). Notably, many SMs also have medicinal or other useful purposes, such as dyes, food additives, and monomers for novel plastics (Alberti et al., 2017). However, we classified the genes in a BGC into biosynthetic genes, further essential genes, and gap genes. The biosynthetic genes encode for enzymes that are directly involved in the biosynthesis of the SM, while the further essential genes encode for transporters (Wang et al., 2014), transcription factors (Derntl et al., 2016), or resistance genes (Schrettl et al., 2010). In contrast, gap genes are not involved in the biosynthesis of the SM despite being co-localized in the BGC (Tai et al., 2018). Both, the biosynthetic genes and the further essential genes are necessary for the biosynthesis of an SM in the native organisms (Anyaogu and Mortensen, 2015). We could use FunOrder to detect these essential genes because they share a similar evolutionary background in many fungal BGCs (Vignolle et al., 2021). The FunOrder method contributes to a better understanding of fungal BGCs by adding another layer of information, which supports researchers in the decision of which genes should be considered for laboratory work.

The obvious major shortcoming of the original FunOrder method is the final manual assessment which prevents full automation and high-throughput analyses. Further, the detection of co-evolved genes may be useful to address a broad range of biological problems related to the molecular coevolution of genes or proteins in all forms of life. Notably, this requires the compilation and integration of suitable proteome databases; for the application in plants or mammals, larger databases will be needed. In this study, we describe an improved version of the method, termed FunOrder 2.0 which overcomes the two mentioned limitations. For an automated detection of co-evolving genes, we determine the optimal number of gene groups in the FunOrder output and then use k-means clustering based on the first three principal components of a PCA. Further, we replace BLAST with the faster DIAMOND tool (Buchfink et al., 2021) to enable the usage of larger databases in the future.

## Materials and methods

### Changes in the workflow

Within the previously developed workflow (Vignolle et al., 2021) (**Figure 1A**), we replaced BLAST (Camacho et al., 2009) with DIAMOND (Buchfink et al., 2021) for the database search (**Figure 1B**). Notably, the BLAST algorithm was kept in the software bundle to extract the sequences from the local database and for an optional remote search of the NCBI database (Altschul et al., 1997). The distance measures obtained after the treeKO algorithm were compiled in matrices, which were used as input for three alternative R-scripts (**Figure 1B**). In all three R-scripts, first, the strict distance matrix and the evolutionary distance matrix are combined into a third distance, the combined distance matrix, as described previously (Vignolle et al., 2021). Next, individual PCAs are calculated for the strict and the combined distance matrices. The three scripts differ in how exactly the co-evolved genes are determined.

The first and second R-scripts aim to determine the co-evolved genes (clusters in the PCA) automatically. To this end, the first three principal components of the PCAs are considered in a k-means clustering approach. In the first R-script, the optimal number of clusters is initially determined by NbClust (Charrad et al., 2014) using 28 indices (**Table 1**). We limited the maximum number of possibly definable clusters to 5 and chose Ward´s minimum variance method based on the Euclidean distance for optimal cluster search within the NbClust function (Murtagh and Legendre, 2014). The second R-script performs a k-means clustering with a preset number of 3 clusters; it is only called as a backup if the prediction of an optimal number of clusters in the first script fails. In both cases, the determined clusters are visualized in a color-coded plot of the first two principal components of the PCAs under “FunOrder_clustering_Rplots_pred.pdf” or “FunOrder_clustering_Rplots_defined.pdf” and as table under “cluster_definition_pred.xlsx” or “cluster_definition_3.xlsx”. FunOrder uses the “protein IDs” of the input file to label the analyzed proteins in the output.

**Table 1:** Indices used to determine the optimal number of clusters.

Index	Reference
Bale index	(Beale, 1969)
Ball index	(Ball and Hall, 1965)
CCC index	(Sarle and SAS Institute, 1983)
CH index	(Caliński and Harabasz, 1974)
C-index	(Hubert and Levin, 1976)
DB index	(Davies and Bouldin, 1979)
Duda index	(Duda and Hart, 1973)
Dunn index	(Dunn, 1974)
Frey index	(Frey and van Groenewoud, 1972)
Friedman index	(Friedman and Rubin, 1967)
Gamma index	(Baker and Hubert, 1975)
Gap index	(Tibshirani et al., 2001)
Gplus index	(Rohlf, 1974; Milligan, 1981)
Hartigan index	(Hartigan, 1975)
KL index	(Krzanowski and Lai, 1988)
Marriot index	(Marriott, 1971)
McClain index	(McClain and Rao, 1975)
Pseudot2 index	(Duda and Hart, 1973)
Ptbiserial index	(Milligan, 1980; Milligan, 1981)
Ratkowsky index	(Ratkowsky and Lance, 1978)
Rubin index	(Friedman and Rubin, 1967)
Scott index	(Scott and Symons, 1971)
SD index	(Halkidi et al., 2000)
SDbw index	(Halkidi and Vazirgiannis, 2001)
Silhouette index	(Rousseeuw, 1987)
Tau index	(Rohlf, 1974; Milligan, 1981)
Tracew index	(Milligan and Cooper, 1985)
Trcovw index	(Milligan and Cooper, 1985)

The third R-script is a revised version of the R-script used in the original FunOrder method (Vignolle et al., 2021). It was simplified by removing unnecessary Euclidean distance calculations and the order of the called functions was rearranged. Now, the strict distance matrix is analyzed before the combined distance matrix. Further, we rearranged the order of the visualizations in the output “FunOrder_Supplementary_Rplots.pdf”. This output consists of heatmaps of the distance matrices, dendrograms of a Ward’s minimum variance clustering of the Euclidean distance within the matrices, and score plots of the first two primary components of the two performed PCA, and must be assessed manually to determine the co-evolved genes (i.e., clusters in the PCA) as described previously (Vignolle et al., 2021).

The software bundle is written in the BASH (Bourne Again Shell) environment and is deposited in the GitHub repository https://github.com/gvignolle/FunOrder (doi: 10.5281/zenodo.6914845). Details on all included scripts can be found in the ReadMe file on the GitHub repository. FunOrder 2.0 requires some dependencies, for details and links to all dependencies please refer to the ReadMe file.

### Control gene clusters

For the evaluation of FunOrder 2.0 and comparison to the original method, we used the same control gene clusters (GC) as in the original study (Vignolle et al., 2021). As benchmark BGCs, we used 30 previously empirically defined BGCs. As negative controls, we used randomly assembled GCs. As a positive control, we used enzymes of conserved metabolic pathways of the primary metabolism. The sequences of all test and control sets are deposited in the GitHub repository https://github.com/gvignolle/FunOrder.

### Calculation of the internal coevolution quotient

“The internal coevolutionary quotient (ICQ) expresses how many genes in a GC or proteins in a protein set are co-evolved according to the previously defined threshold for strict and combined distances within the distance matrices of an analyzed GC (or protein set).” (Vignolle et al., 2021) The ICQ values were calculated using Equation 1.


$$IDQ=1-\left\{\frac{g}{2*\left[d*(d-1)\right]}\right\}$$

**Equation 1**. ICQ = internal coevolutionary quotient; g = number of strict distances< 0.7 and combined distances<= (0.6 * max value of the combined distance matrix) in all matrices; d = number of genes in the GC (Vignolle et al., 2021).

### Performance evaluation

Similar to the original method we analyzed 30 empirically characterized BGCs to evaluate the ability of FunOrder 2.0 to identify presumably co-evolved essential genes (as defined in **Table S3**) and to distinguish them from so-called gap genes and genes outside of the defined BGC borders. The genes clustering with the core enzyme(s) were considered as “detected”. As previously described “we counted the total number of (1a) detected essential genes or (1b) detected biosynthetic genes, (2a) not detected essential genes or (2b) not detected biosynthetic genes, (3) detected gap and extra genes, and (4) not detected gap or extra genes in all BGCs, and defined (1a or 1b) as true positives (TP), (2a or 2b) as false negatives (FN), (3) as false positives (FP), and (4) as true negatives (TN)” (Vignolle et al., 2021), which were finally used as input for a stringent statistical analysis (Vignolle et al., 2021).

## Results

### Integration of the DIAMOND algorithm

The first major improvement of the FunOrder method was the integration of the DIAMOND algorithm (Buchfink et al., 2015; Buchfink et al., 2021) for searching the proteome database instead of the previously used BLAST algorithm (Camacho et al., 2009) (**Figure 1**). This change will allow the usage of larger databases in FunOrder 2.0, since DIAMOND is as sensitive as BLAST, but is faster and is adapted to larger databases (Buchfink et al., 2021). With DIAMOND the run time of the first step in the FunOrder pipeline was reduced significantly. For instance, the database search for the lovastatin BGC of *Aspergillus terreus* (lov) (Mulder et al., 2015) took 1 m 25 sec using the original FunOrder method, and 45 sec real-time using FunOrder 2.0. This difference will of course be more pronounced when a larger database is used.

**Figure 2:**
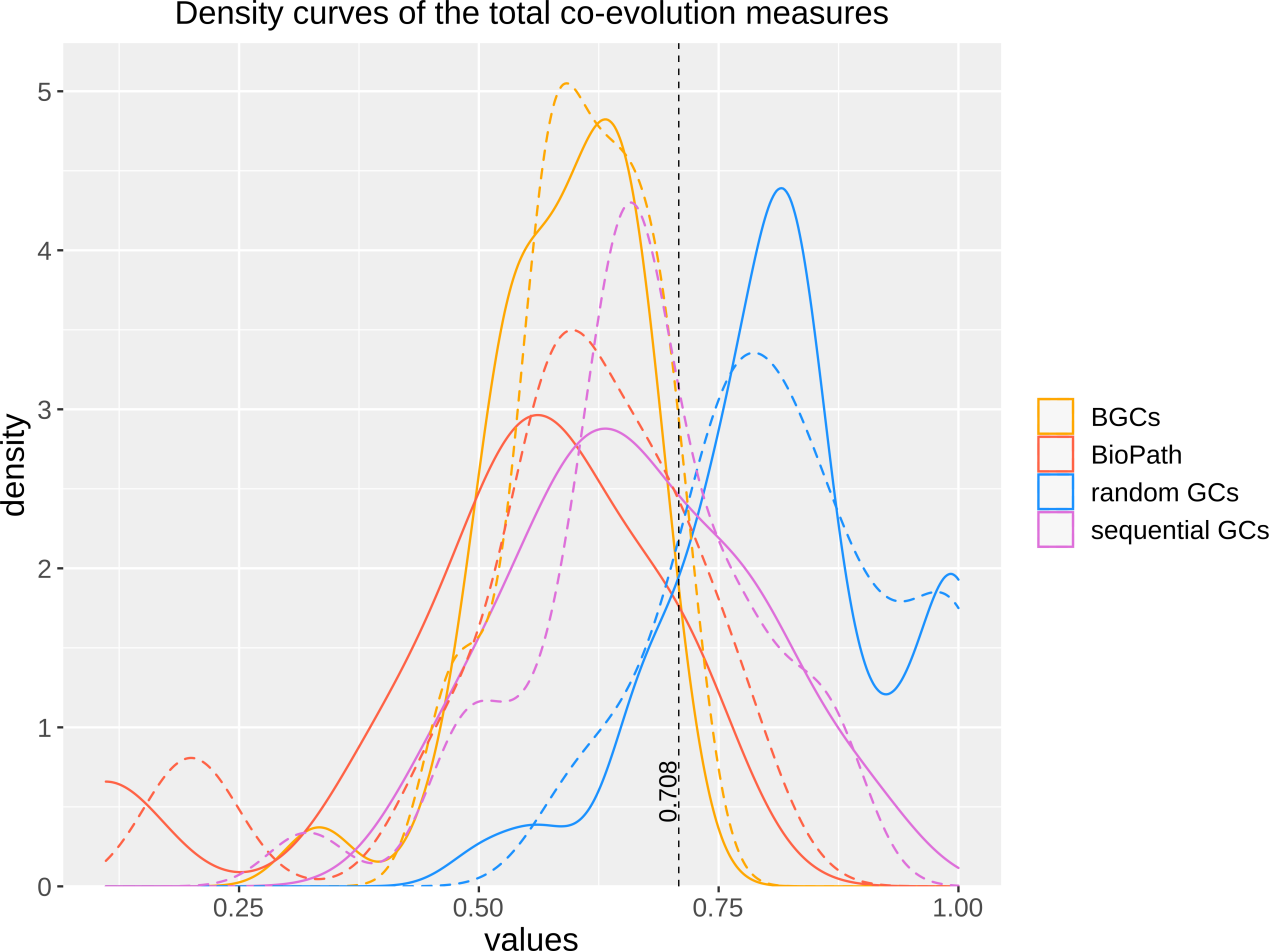
Kernel density plot of the ICQ values for co-evolutionary linked enzymes of different control sets comparing the original FunOrder method (dashed lines) and FunOrder 2.0 (solid lines). BGCs, previously empirically characterized fungal BGCs; BioPath, protein sets of conserved biosynthetic pathways of the primary metabolism; random GCs, randomly assembled protein sets from 134 fungal proteomes; sequential GCs, co-localized genes from random loci of different ascomycetes.

To test, whether the integration of DIAMOND might have altered the ability of FunOrder to detect coevolution, we analyzed the same control gene clusters (GCs) we had previously used to evaluate the original FunOrder method (Vignolle et al., 2021) and calculated the internal coevolution quotient (ICQ). The ICQ expresses how many genes in a gene cluster are detected as coevolutionary linked and is calculated subsequently to the treeKO comparison (**Figure 1**). Since no other changes have been introduced until this point in the workflow, the ICQ values are a feasible way to compare BLAST and the DIAMOND software. We found only marginal differences between the original FunOrder method (using BLAST) and FunOrder 2.0 (using DIAMOND) (**Table S1**). For visualization, we compared the ICQ results in a kernel density plot (**Figure 2**). Therein, the curve for the ICQs of the positive control GCs (BioPath in **Figure 2**) slightly shifted to the left (higher internal coevolution) compared to the original method, while the curve for the negative control GCs (random GCs in **Figure 2**) slightly shifted to the right (lower internal coevolution). These results indicate that DIAMOND might be better suited than BLAST within the FunOrder method, as the usage of DIAMOND resulted in a better distinction of the positive and negative control GCs. The curve for the sequential GCs was flattened and broadened compared to the original curve (**Figure 2**), which can also be explained by the assumed better performance of DIAMOND in this workflow. As the sequential GCs are random loci from different ascomycetes (Vignolle et al., 2021), they contain random numbers of co-evolved and independently evolved genes. Consequently, the usage of DIAMOND lowers the ICQ for GCs containing many co-evolved genes and raises the ICQ for GCs with many independently evolved genes compared to the original FunOrder method. This results in the detection of simultaneously more and less coevolution in all sequential GCs and therefore a flattening of the curve in **Figure 2**. For the benchmark BGCs, we could not observe a drastic change in the height or position of the curve, but a change in the shape with no significant differences in the variance and the mean (**File S1**). However, the changes in the curves of the random GCs and the BGCs resulted in a new point of intersection (0.708), which should be considered in the final assessment of fungal BGCs. In BGCs with an ICQ above this threshold, no statistically relevant internal co-evolution could be determined, which might indicate an evolutionary background other than co-evolution.

### Automated cluster definition

As mentioned, a major limitation of the original FunOrder method was the need for a manual assessment of the output, during which the proteins are grouped into clusters based on different data visualizations (**Figure 1A**) (Vignolle et al., 2021). In this regard, only the first two components could be considered for graphical and practical reasons (two-dimensional score plot). To solve these problems, we integrated two R scripts for the automatic definition of co-evolved protein groups (or clusters) (**Figure 1B**). To consider a higher proportion of the underlying data for the clustering, the two R scripts use the first three principal components of the PCA of the strict and the combined distance matrices as input (**Figure 1B**) and group the proteins by k-means clustering. We reasoned, that considering further components would reintroduce data noise.

The first R-script for automated clustering initially determines the optimal number of gene clusters within the first three principal components of the PCAs using the R Package NbClust (Charrad et al., 2014). This package uses different indices and varies the number of clusters, distance measures, and clustering methods to determine the optimal number of clusters in a data set based on the majority rule. If the prediction of the optimal number of clusters fails, the third script with a predefined number of clusters is called as a backup. The prediction of the optimal number of clusters might fail for instance if the majority rule cannot be applied. As we aim to distinguish biosynthetic, further essential, and gap genes in fungal BGCS, we predefined the number of clusters to 3. Regardless of the script used, the final output is an excel file (**Table S2**) and a color-coded visualization of the PCA (**File S2**). A revised version of the original R-script used in FunOrder (Vignolle et al., 2021) was kept as an option in the software bundle.

To test how this automated cluster definition compares to the previously performed manual cluster definition, we analyzed the same 30 BGCs as in our previous study. To observe only the influence of the automated cluster definition, we kept the BLAST tool for the initial database search still in place (**Figure 1**). Then, we compared the obtained results to those of the previously performed manual analyses (Vignolle et al., 2021) (**Table 2** and **Table S3**). In only 5 out of the tested 30 BGCs, the same results were obtained (**Table S3**). In 15 BGCs, the automated cluster definition missed at least one biosynthetic or further essential gene in comparison to the manual assessment, but it could detect more of these essential genes in 5 BGCs. Regarding the gap and extra genes, the automated cluster definition returned fewer false positives than the manual assessment in 12 BGCs but found more in 4 BGCs. In summary, the automated cluster detection appeared to be more stringent than the manual assessment method, which led to slightly reduced sensitivity but enhanced selectivity (see **Table S3** for a detailed statistical analysis).

**Table 2:** Number of BGCs in which the automated cluster detection (in combination with BLAST or DIAMOND) delivered the same, better, worse, or different results for the given gene categories compared to the manual method.

	BLAST/DIAMOND			
	Same	Better	Worse	Different genes
biosynthetic genes	16/17	4/4	10/9	-/-
further essential genes	11/11	3/3	5/5	-/-
gap genes	13/13	8/8	3/3	3/3
extra genes	16/16	5/5	2/3	1/-

Next, we tested the simultaneous influence of DIAMOND and the automated clustering on the overall performance of FunOrder 2.0 during the analysis of fungal BGCs. To this end, we performed the same comparative analysis of the benchmark BGCs as described above. The results were very similar to the automated analysis using the BLAST analysis (**Table 2** and **Table S3**). In a few cases, the usage of DIAMOND improved the automated cluster definition compared to BLAST, but it remained still more stringent than the manual assessment (**Table 2** and **Table S3**). Fewer biosynthetic genes or further essential genes were detected in 13 of the 30 BGCs by FunOrder 2.0, but also fewer gap or extra genes in 12 BGCs (**Table 2** and **Table S3**). Yet, FunOrder 2.0 clustered more genes together than the original method in other BGCs - to be precise, more essential genes were detected in 5 BGCs and more gap or extra genes in 4 BGCs compared to the original method (**Table S3**). The overall enhanced stringency reduced the sensitivity slightly (**Table 3**) but also improved several statistic measures, including specificity, precision, and the normalized Matthew correlation coefficient (**Table 3**, in bold). To test if the observed differences have a significant impact on the overall applicability of FunOrder 2.0 in fungal BGCs, we further compared the percentages of correctly identified genes in each BGC between the original FunOrder and FunOrder 2.0 (**Tables S3**) in an ANOVA (**File S1**) and found no significant difference. Taken together, we conclude that the introduced changes allow the detection of coevolution between different proteins with an enhanced stringency and precision compared to the original method and that FunOrder 2.0 can be used to identify essential genes in fungal BGCs.

**Table 3:** Performance comparison of the original FunOrder (Vignolle et al., 2021) and FunOrder 2.0 for detecting relevant genes in fungal BGCs.

	FunOrder essential genes	FunOrder 2.0 essential genes	FunOrder biosynthetic genes	FunOrder 2.0 biosynthetic genes
Sensitivity	0.6349	0.6266	0.6615	0.6564
Specificity	0.8112	**0.8541**	0.8112	**0.8541**
Precision	0.7766	**0.8162**	0.7457	**0.7901**
Negative Predictive Value	0.6823	**0.6886**	0.7412	**0.7481**
False Positive Rate	0.1888	**0.1459**	0.1888	**0.1459**
False Discovery Rate	0.2234	**0.1838**	0.2543	**0.2099**
False Negative Rate	0.3651	0.3734	0.3385	0.3436
Accuracy	0.7215	**0.7384**	0.7430	**0.764**
F1 Score	0.6986	**0.7089**	0.7011	**0.7171**
Matthews Correlation Coefficient	0.4524	**0.4926**	0.4797	**0.5242**
Normalized Matthews Correlation Coefficient	0.7262	**0.7463**	0.73985	**0.7621**
No-information error rate ni	0.5084	0.5084	0.5444	0.5444

Improved statistical measures are highlighted in bold.

The automation of the cluster detection in FunOrder 2.0 prevents user bias and improves the overall speed. The analysis of the lovastatin BGC of *Aspergillus terreus* (lov) (Mulder et al., 2015) with 17 genes, took 1 h 19 m 48 sec real-time using 22 threads on an Ubuntu Linux system with 128 GB DDR4 RAM with the original FunOrder (excluding manual cluster definition) and 1 h 19 m 58 sec real-time with FunOrder2. Notably, the runtime for FunOrder 2.0 includes already the automated detection and grouping of co-evolving genes, which takes an experienced user additional 30 - 45 minutes during the original method.

## Discussion

The integration of the DIAMOND tool and the automated detection of co-evolved genes improved the run time and total analysis time, allowing high throughput analysis of protein sets and GCs without the risk of user bias. In general, the automated cluster definition appears more stringent than the manual assessment, which resulted in improved specificity and precision and a slightly reduced sensitivity during the analysis of fungal BGCs by FunOrder 2.0 compared to the original method. In summary, we consider the integration of a fully automated cluster definition a major improvement, as the advantages (speed, reproducibility, precision) outweigh the slightly reduced sensitivity.

Recently, Steenwyk et al. established another method for the calculation of co-evolution. The comprehensive PhyKIT tool calculates the covarying evolutionary rates of orthologous genes using correlation of relative evolutionary rates between orthologues (CovER function) (Steenwyk et al., 2021). This approach compared the lengths of shared branches in gene trees corrected by the species tree. Later, this method was used to calculate a network of coevolving orthologues as complementary approach to genetic network analyses by the same group (Steenwyk et al., 2022). Therein, a set of 2408 orthologous genes from 332 species of budding yeasts are compared and assessed. It is difficult to directly compare the CovER function of PhyKit (Steenwyk et al., 2021) and FunOrder 2, as they rely on a different methodological approach. PhyKit relies on a direct comparison within an input data set, whereas FunOrder 2 uses a database to infer co-evolution of a small set of input protein sequences. We speculate that PhyKit might obtain more precise results with higher resolution, especially when large input sets are used, whereas FunOrder 2 might be faster and more suitable for the analysis of small input sets and data from species without a lot of sequenced relatives.

As demonstrated, FunOrder 2.0 can be used to determine the essential genes in fungal BGCs, but this is not the only potential application of FunOrder 2.0. As protein coevolution can be used to predict protein-protein interactions and biosynthetically linked enzymes (Ochoa and Pazos, 2014; Vignolle et al., 2021), FunOrder 2.0 may be used to answer many different research questions. Potentially, even complete fungal genomes might be assessed by our method. It is also exciting to speculate if and how FunOrder may be used in other clades of life. A limitation in this regard might be the maximum number of predicted clusters. NbClust limits the number of potential clusters to 15, we further lowered this number to 5 for the analysis of BGCs. This problem might be circumvented by an arbitrary definition of the number of clusters or by consecutive FunOrder analyses, in which a large output cluster is used as input for a follow-up analysis.

FunOrder 2.0 is provided with a database of ascomycete proteomes and can therefore be used for the detection of coevolution of proteins in this fungal division. For other divisions, classes, or even kingdoms, a suitable new proteome database must be compiled and tested. As mentioned, the integration of the DIAMOND tool enables the integration of larger databases. However, at least 25 different proteomes must be used, because the phylogenetic trees are calculated with a maximum of 20 homologous sequences. Naturally, the proteomes should be of high quality (best RNASeq derived). The proteomes shall be equally distributed among the taxonomic rank to be analyzed but also take the size of the different subordinate ranks into consideration. Put differently, if a division contains 4 small classes, and two large classes, the database should contain proteomes of all six classes, but more from the larger classes than from the smaller classes. The database shall be a representative sample of the phylogenetic group to be analyzed. This also means that highly diverse phylogenetic groups need to be over-represented in comparison to evolutionary uninventive clades. Further, evolutionary outliers and special clades shall be considered in the database design. For instance, if a phylum contains a family that is the only member of its class, the user needs to decide whether that family shall be part of the proteome database at all, depending on the size and importance of the family. If the family shall be considered, several proteomes need to be included in the database, otherwise, the evolutionary distances of the tested proteins might be too large to be successfully evaluated by FunOrder. Any new database must be tested thoroughly according to the procedure we described previously (Vignolle et al., 2021). This means, that suitable test gene clusters must be compiled and that meaningful thresholds for the strict and combined distance should be defined. If possible, a test set of target gene clusters should be analyzed and compared to previous results. Please refer to our previous study on how we tested the ascomycete database, determined the thresholds, and tested the applicability of FunOrder for the detection of essential genes in BGCs in ascomycetes (Vignolle et al., 2021). A possible shortcut in this procedure might be determining the thresholds of strict and combined distance *via* threshold optimizing (best-obtained distinction of positive and negative control gene clusters). Please also refer to the technical guidelines for construction and integration of the database at the GitHub repository https://github.com/gvignolle/FunOrder.

## Data availability statement

The FunOrder tool, the relevant database, the sequences, and the FunOrder output of the negative control GCs and the positive control BGCs are available in the GitHub repository (https://github.com/gvignolle/FunOrder). We have also used Zenodo to assign a DOI to the repository: 10.5281/zenodo.6914845.

## Author contributions

GV: conceptualization, data curation, formal analysis, investigation, methodology, software, validation, visualization, and writing – original draft preparation; RM: resources, writing – review and editing; AM-A: resources, and writing – review and editing; CZ: conceptualization, funding acquisition, methodology, validation, project administration, supervision, visualization, and writing – original draft preparation. All authors contributed to the article and approved the submitted version.

## Funding

This study was supported by the Austrian Science Fund (FWF, https://www.fwf.ac.at/) [P 34036 to CZ] and TU Wien (https://www.tuwien.at/) [Ph.D. program TU Wien bioactive]. The funders had no role in the study design, data collection, and analysis, decision to publish, or preparation of the manuscript.

## Conflict of interest

Author GV was employed by the company AIT Austrian Institute of Technology GmbH. The remaining authors declare that the research was conducted in the absence of any commercial or financial relationships that could be construed as a potential conflict of interest.

## Publisher’s note

All claims expressed in this article are solely those of the authors and do not necessarily represent those of their affiliated organizations, or those of the publisher, the editors and the reviewers. Any product that may be evaluated in this article, or claim that may be made by its manufacturer, is not guaranteed or endorsed by the publisher.

## Supplementary material

File S1 ANOVA for the percentage of correctly detected genes detected by FunOrder and FunOrder 2.0, respectively.

File S2 FunOrder 2.0 output of the Lovastatin BGC from *A. terreus* (lov).

Supplementary Table 1 ICQ values of protein sets of conserved metabolic pathways of the primary metabolism (BioPath), sequential GCs, and random GCs used in this study.

Supplementary Table 2 FunOrder 2.0 output of the Lovastatin BGC from *A. terreus* (lov).

Supplementary Table 3 Results for the analyses of benchmark BGCs using different versions of the FunOrder method.
