# Supplementary material for: FunOrder 2.0 – a method for the fully automated curation of co-evolved genes in fungal biosynthetic gene clusters
Source: Front Fungal Biol. 2022 Oct 25;3:1020623. doi: 10.3389/ffunb.2022.1020623 (PMC10512238; doi:10.3389/ffunb.2022.1020623)
Supplement: File S1 — ANOVA for the percentage of correctly detected genes detected by FunOrder and FunOrder 2.0, respectively. [file DataSheet_1.pdf]

Statistical analysis of the relative discovery rates of essential or biosynthetic genes

All the statistical tests were performed in the R environment. The Shapiro-Wilk test used below was used to check for the normality of the percentages of detected essential or biosynthetic genes, as previously defined, between the FunOrder 1 and FunOrder 2 output (Table 1). Normality assumptions underlie outlier detection hypothesis tests. If the p-value is above the set alpha significance value (0.01) then the null hypothesis is not discarded. In other words, it can be considered a normal distribution.

Table 1 Shapiro-Wilk normality tests.

**data set**	**p-value**
FunOrder 1 – % essential genes	0.2236
FunOrder 1 – % biosynthetic genes	0.007389
FunOrder 2 – % essential genes	0.1738
FunOrder 2 – % biosynthetic genes	0.05362

Table 2 Levene's Test for Homogeneity of Variance (center = median) performed on the percentages of detected essential or biosynthetic genes, as previously defined, between the FunOrder 1 and FunOrder 2 output.

	**Df**	**F value**	**Pr(>F)**
**performance data sets**	3	0.4007	0.7527

From the output in table 2, it can be seen that the p-value was not less than the significance level of 0.05. This means that there was no evidence to suggest that the variance is statistically significantly different for the data sets. Levene’s test is an alternative to Bartlett’s test when the data is not normally distributed.

Table 3 Computed one-way ANOVA test the analysis of variance performed on the percentages of detected essential or biosynthetic genes, as previously defined, between the FunOrder 1 and FunOrder 2 output.

	**Df**	**Sum Sq**	**Mean Sq**	**F value**	**Pr(>F)**
**performance data sets**	3	1443	481.0	0.853	0.468
**Residuals**	116	65447	564.2		

The output in table 3 includes the columns F value and Pr(>F) corresponding to the p-value of the test. As the p-value is higher than the significance level 0.05, we could conclude that there are no significant differences between the percentages of relative discovery rate of essential or biosynthetic genes in the model summary between FunOrder 1 and FunOrder 2.

We further compared the internal co-evolution quotient (ICQ) of both the FunOrder 1 and FunOrder 2 output for the BGCs. A F-test resulted in F = 0.76644 with a p-value = 0.4783, since the p-value is above the significance level 0.05 we could conclude that there is no significant difference between the variances in the two sets of ICQs. We continued with a two sided two sample t-test to compare the means of the two datasets. The t-test had a p-value of 0.2682, since we obtained a p-value greater than 0.05 we can conclude that means of the two datasets have no significant difference and can be regarded as equal.
