# Supplementary figures and images for: FunOrder 2.0 – a method for the fully automated curation of co-evolved genes in fungal biosynthetic gene clusters
Source: Front Fungal Biol. 2022 Oct 25;3:1020623. doi: 10.3389/ffunb.2022.1020623 (PMC10512238; doi:10.3389/ffunb.2022.1020623)

# Score plot of PCA of strict distance

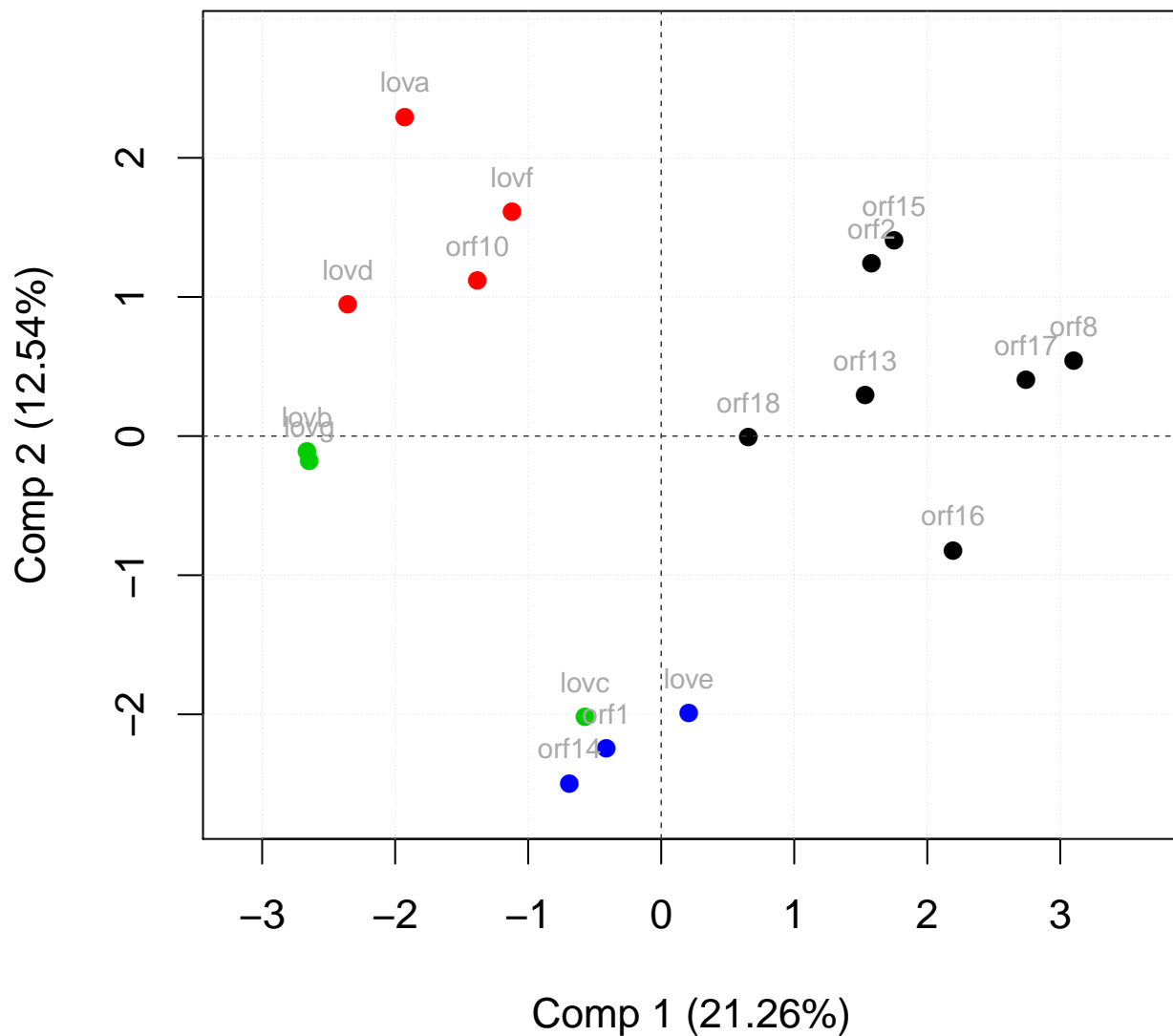

# Score plot of PCA of combined distance

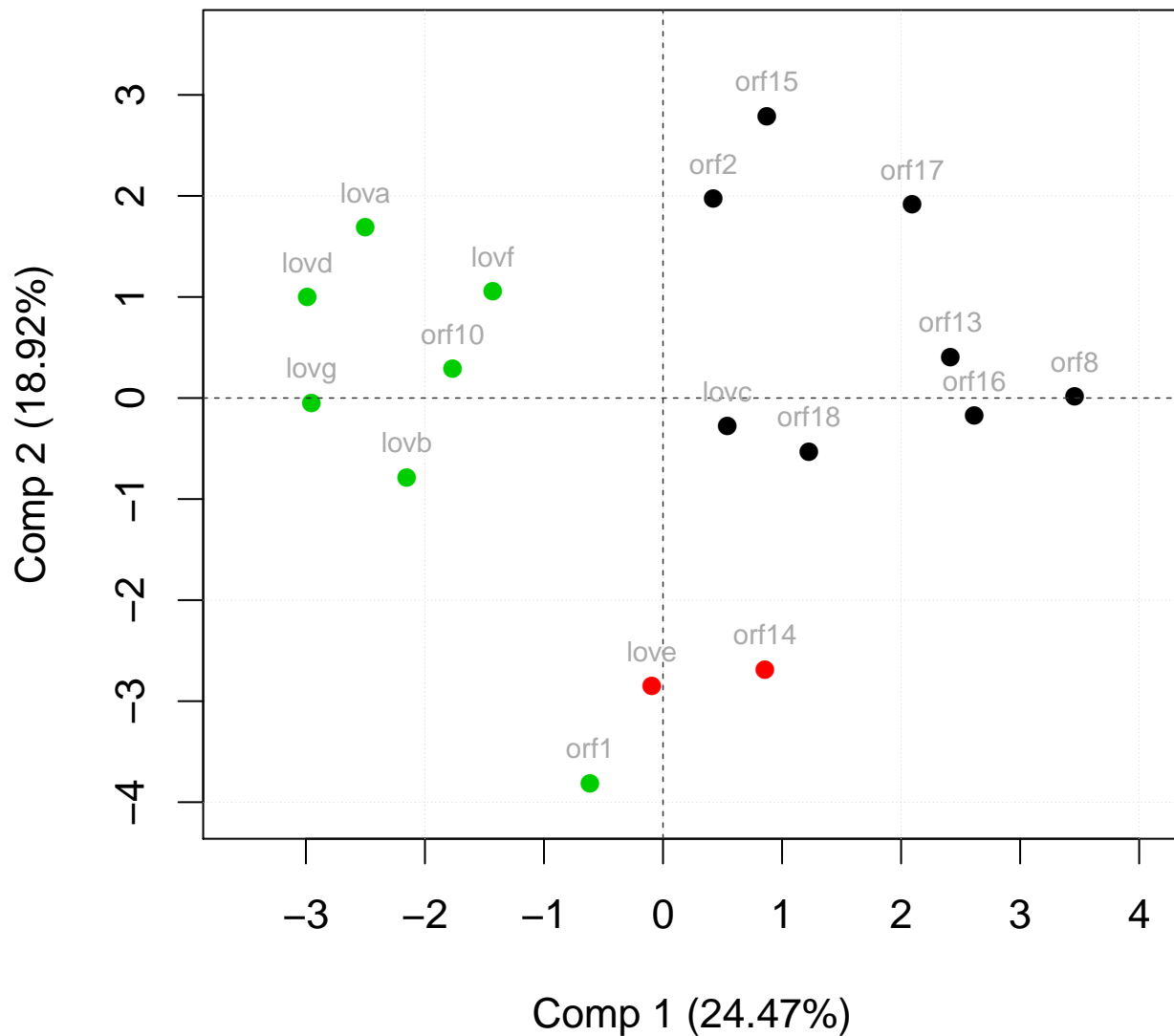

Supplement: File S2 — FunOrder 2.0 output of the Lovastatin BGC from A. terreus (lov). [file DataSheet_2.pdf]
